# 

**Published:** 1898-04-09

**Authors:** 


**Notices of Births, Deaths, and Marriages** are inserted in this column at a charge of 2s. 6d. for an announcement not exceeding four lines.

### NOTICE TO CORRESPONDENTS.

All MS., Letters, Books for Review, and other matters intended for the Editor should be addressed The Editor, "The Hospital," The Hospital Building, 28 & 29, Southampton Street, Strand, W.C.
The Editor cannot undertake to return rejected MS., even when accompanied by stamped directed envelope.

**NOTICE.—Vol. XXIII. of "The Hospital" (October, 1897, to March, 1898), in handsome cloth binding, will shortly be on sale at the Office of this Journal, price 7s. 6d. Cases for binding the Numbers contained in this Volume, 1s. 6d. Index to Vol. XXIII., 2½d., post free.**

**The Monthly Part for April is now ready. Price 6d., by post 8d.**

**Notice to Advertisers and Subscribers.**

All Advertisements, Orders for Copies of The Hospital, and Business Communications should be addressed to **THE MANAGER, (Not to The Editor),** The Hospital, The Hospital Building, 28 & 29, Southampton Street, Strand, London, W.C.

The Cost of Subscription, post free, is as follows:—Per week, 2½d. per quarter, 2s. 8d.; per half-year, 5s. 3d.; per annum, 10s. 6d. Foreign:—Per annum, 15s. 2d.; payable, in all cases, in advance.

### BOOKS RECEIVED.

H. K. Lewis.

"Inflammation of the Bladder and Urinary Fever." By C. Mansel Moullin, M.D., F.R.C.S.

The Rebman Publishing Company.

"A Clinical Text Book of Surgical Diagnosis and Treatment." By J. W. Macdonald, M.D.

Bailliere, Tindall, and Cox.

"Respiratory Exercises in the Treatment of Disease. By Henry Campbell, M.D.

### The Student of Medicine.

### APPOINTMENTS.

D. H. Attfield, M.A., M.B.Camb., appointed Medical Officer of Health to the Watford Urban District Council. R. A. Bennett, M.B.Lond., M.R.C.S., L.R.C.P., appointed Resident Medical Officer of the Manchester Hospital for Consumption and Diseases of the Chest. Dr. Bishop, appointed Medical Officer for the Barham District of the Bridge Union. Eleanor C. Bond, M.D.Brux., L.S.A., appointed House Surgeon to the Belgrave Hospital for Children. Dr. Brown, appointed Medical Officer for the No. 1 District of the Hackney Union. J. S. Chater, M.B.Lond., M.R.C.S., L.R.C.P., appointed Senior House Surgeon to the Royal Surrey County Hospital, Guildford. M. Coates, M.D., F.R.C.S.Eng., L.S.A., Retired Deputy Inspector-General, R.N., appointed House Surgeon to the Philanthropic Society. B. Dyball, M.B., B.S.Lond., F.R.C.S., L.R.C.P., appointed Resident Surgical Officer to the General Infirmary, Leeds. R. W. J. Ewart, appointed Medical Officer to the Masham Urban District Council. A. D. Forbes, M.B.Aberd., appointed Medical Officer for the Seventh District (Horsmonder) of the Tonbridge Union. J. F. Gairdner, M.B., C.M.Glasg., M.R.C.S.Eng., L.R.C.P.Lond., appointed Resident Casualty Officer to the General Infirmary, Leeds. H. W. Greatbach, M.B., and C.M.-Edin., appointed Medical Officer of Health for the Burgh of Montrose. J. H. Horling, L.S.A., appointed Medical Officer of Health to the Bideford Rural District Council. F. Jaffrey, F.R.C.S.Eng., appointed Assistant Surgeon to the St. George's Hospital. J. H. Joyce, M.B., appointed Medical Officer for the No. 2 District of the Ashby-de-la-Zouch Union. — Lawton, appointed Assistant Medical Officer to the Workhouse of the Edmonton Union. R. R. W. Logan, M.R.C.S.Eng., L.S.A., appointed Medical Officer for the No. 3 District of the Ashby-de-la-Zouch Union. J. C. Muir, B.A., M.B., B.C.Cantab, M.R.C.S.Eng., L.R.C.P.Lond., appointed Junior Resident Medical Officer of the Manchester Children's Hospital. C. J. Myers, M.R.C.S. and L.S.A., appointed Medical Officer of Health to the Louth Rural Sanitary Authority. G. Peterkin, M.B., C.M.Edin, appointed Medical Officer of Health and Police Surgeon for Forfar. C. W. Suckling, M.D.Lond., appointed Consulting Physician to the Queen's Hospital, Birmingham. J. Trant, L.R.C.P.I., L.R.C.S.I., J.P., appointed Medical Attendant to Commercial Cable Company's Staff, Waterville, and Medical Officer, Darrynane Dispensary District. H. B. Woodcock, M.B., Ch.B.Vict., appointed Assistant Medical Officer to the Salford Union Infirmary.

